# CtACO1 Overexpression Resulted in the Alteration of the Flavonoids Profile of Safflower

**DOI:** 10.3390/molecules24061128

**Published:** 2019-03-21

**Authors:** Yanhua Tu, Beixuan He, Songyan Gao, Dandan Guo, Xinlei Jia, Xin Dong, Meili Guo

**Affiliations:** 1 Department of Pharmacognosy, College of Pharmacy, Second Military Medical University, Shanghai 200433, China; tutuxiaohutu-1988@163.com (Y.T.); 18721809513@163.com (B.H.); danna_guo0810@163.com (D.G.); 13916087394@163.com (X.J.); 2 Chemical Experiment Teaching Center, College of Pharmacy, Second Military Medical University, Shanghai 200433, China; sy_gaosmmu@163.com

**Keywords:** safflower (*Carthamus tinctorius* L.), floral dipping method, CtACO1 overexpression, flavonoid accumulation

## Abstract

Background: Flavonoids with various structures play a vital role in plant acclimatization to varying environments as well as in plant growth, development, and reproduction. Exogenous applications of ethylene and 1-aminocyclopropane carboxylic acid (ACC), could affect the accumulation of flavonoids. Very few attempts have been made to investigate the effect of 1-aminocyclopropane carboxylic acid oxidase (ACO), a unique enzyme that catalyzes ACC to ethylene, on genes and metabolites in the flavonoid biosynthetic pathway. In this study, two ACOs in safflower (CtACOs) were cloned, and then transgenic safflower with overexpressed CtACO1 was generated through the Agrobacterium-mediated floral dipping method. Results: CtACO1 and CtACO2 were both characterized by the 2-oxoglutarate binding domain RxS and the ferrous iron binding site HxDxnH as ACOs from other plants. However, the transcript levels of CtACO1 in flowers at stages I, II, III, and IV were all higher than those of CtACO2. At the cellular level, by using electroporation transformation, CtACO1 was found to be localized at the cytomembrane in onion epidermal cells. CtACO1 overexpression had varying effects on genes involved in the ethylene and flavonoid biosynthetic pathways. The metabolites analysis showed that CtACO1 overexpression lines had a higher accumulation of quercetin and its glycosylated derivatives (quercetin 3-β-d-glucoside and rutin). In contrast, the accumulation of quinochalcones (hydroxysafflor yellow A and carthamin), kaempferol glycosylated derivatives (kaempferol-3-*O*-β-rutinoside and kaempferol-3-*O*-β-d-glucoside), apigenin, and luteolin in CtACO1 overexpression lines were decreased. Conclusion: This study confirmed the feasibility of applying the floral dipping method to safflower and showed a novel regulatory effect of CtACO1 in the flavonoid biosynthetic pathway. It provides hypothetical and practical groundwork for further research on regulating the overall metabolic flux of flavonoids in safflower, particularly hydroxysafflor yellow A and other quinochalcones, by using appropriate genetic engineering strategies.

## 1. Introduction

Flavonoids originating from phenylalanine and acetate metabolism are extensively scattered across most plants in nature [1]. Because of the significance of flavonoid content in biology, ecology, and health maintenance, exploring their alteration has attracted much scientific attention over the past years. Several attempts have been made to improve the flavonoid content in plants through the application of phytohormones, which may stimulate the expression of plant defense genes, thereby affecting the accumulation of secondary metabolite production of plants to some degree. As a gaseous phytohormone, ethylene regulates various processes of plant growth, development, and stress responses and acts as a signaling molecule in the interplay with the environment [2,3]. Previous studies have shown that exogenous applications of ethylene and its precursor, 1-aminocyclopropane carboxylic acid (ACC), could affect flavonoid accumulation to some extent, which may be regulated by the ethylene receptor 1(ETR1) and ethylene insensitive 2 (EIN2) proteins [4,5]. In the ethylene biosynthesis pathway, 1-aminocyclopropane carboxylic acid oxidase (ACO) participates in the last step of ethylene biosynthesis and catalyzes ACC to ethylene [6,7]. As a unique enzyme implicated only in the ethylene biosynthesis pathway, ACO has been obtained from many plants such as *Solanum lycopersicum* [8], *Malus domestica* [9], *Glycine max* [10], and *Gossypium hirsutum* [2]. A previous study showed that acetylsalicylic acid inhibits ethylene production by inhibiting ACO activity [11]. RNA interference in 1-aminocyclopropane-1-carboxylic acid oxidase (*ACO1* and *ACO2*) gene expression has also been reported to prolong the shelf life of *Eksotika papaya* fruit [12]. These studies showed that ACO was related to the physiological and biochemical changes in plants. However, to our knowledge, the effect of ACO on flavonoid accumulation or on the flavonoid metabolic pathway remains uninvestigated.

In addition, advances in molecular biotechnology have produced another way to modify content accumulation in plants. Specifically, the transgenic approach has provided novel possibilities for overproducing or silencing enzymes involved in the metabolic pathway, leading to the preferential accumulation or absence of some compounds in a target organ or tissue [13]. For example, overexpression of *Cs*F3′5′H resulted in the production of new delphinidin derivatives in the corollas of transgenic tobacco plants, and increased the content of cyanidin derivatives, and produced deeper and redder flowers, in transgenic plants [14]. Overexpression of a grapevine R2R3-MYB factor in tomato affected the flower morphology, and the flavonoid and terpenoid metabolism [15]. Safflower (*Carthamus tinctorius* L.) is a versatile oilseed crop with several desirable features. Apart from being cultivated as an oilseed crop, it has also emerged as a medicinal plant that has been used to treat a broad range of ailments in traditional Chinese medicine, such as coronary heart disease, cerebrovascular diseases, gynecologic diseases, and hypertension [16,17]. Thus far, it has also been developed as a platform to generate the production of transgenic safflower, such as modified gamma-linolenic acid, human insulin and apolipoprotein. In in vitro conditions, safflower shoots are impressionable to hyperhydration and are frequently poor at forming roots, which often fail to survive when transferred from tissue culture media to soil, thus impeding the application of tissue culture approaches to safflower [18]. However, an alternative method of safflower transformation, called in plant transformation mediated by Agrobacterium, has emerged and a generation of stably transformed plants and progeny, which was then improved through grafting, was described in [19]. As a flowering plant, safflower, especially its tubular flowers, has yielded a striking variety of flavonoids such as hydroxysafflor yellow A, kaempferol-3-*O*-β-rutinoside, and kaempferol-3-*O*-β-d-glucoside, which are known to be the predominant active contents though which safflower displays its potency. Nevertheless, no previous studies have explored the modification of flavonoids in safflower through transgenic approaches.

The present work first describes the isolation and characterization of CtACOs from safflower. The open reading frame of CtACO1 is then subcloned into an overexpression vector for transforming safflower through the Agrobacterium-mediated floral dipping method and for determining the subcellular localization of CtACO1. Simultaneously, the transcriptome expression patterns of selected genes involved in the ethylene and flavonoid metabolic pathways in transgenic safflower are analyzed. Moreover, the accumulation of flavonoids in different organs is quantified. Overexpression of CtACO1 in transgenic safflower is found to lead to widespread changes in flavonoids, supporting the role of CtACO in regulating flavonoid metabolism and suggesting a metabolic engineering strategy for improving the accumulation of flavonoids in safflower.

## 2. Results

### 2.1. Discovery and Characterization of CtACOs from the Transcription Profiling Database of Safflower

**Figure 1:**
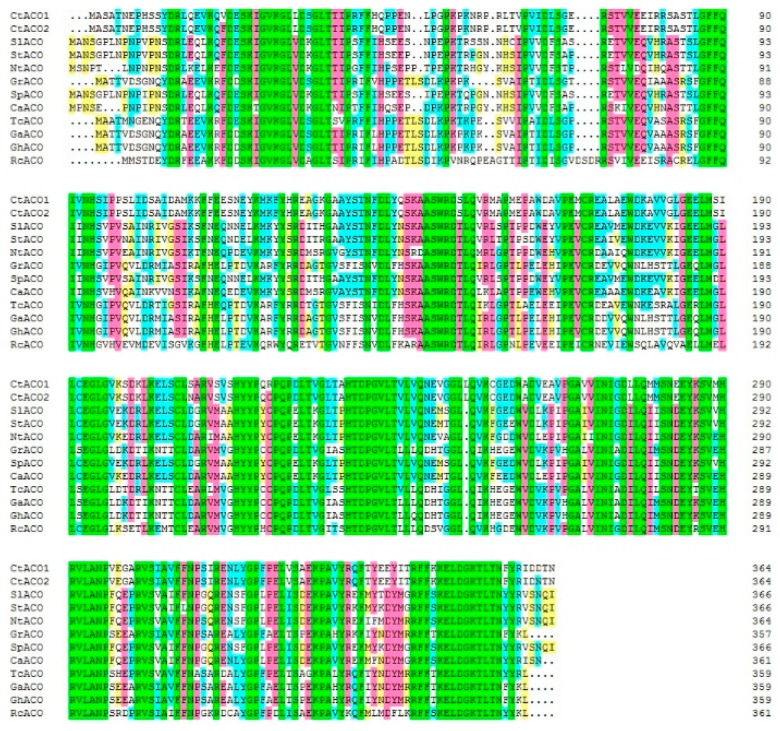
Alignment of CtACO1 and CtACO2 with ACOs（1-aminocyclopropane carboxylic acid oxidase） from other species. Genbank accession numbers for the proteins in the alignment are as follows: *Sl*ACO1(XP_004236932), *St*ACO11 (XP_006355064), *Sp*ACO 4(XP_015074312), *Ca*ACO4(XP_016572720), *Tc*ACO (XP_007045252), *Ga*ACO(XP_017612243), *Gh*ACO(XP_016665668), *Rc*ACO(XP_002525989), CtACO1 (KU291182) and CtACO2 (KU291190). The sequences were aligned using DNAMAN 8.0 software (Lynnon Biosoft, San Ramon, CA, USA).

Flower transcriptome sequencing was carried out to study the functional genes in safflower. The expressed sequence tags (ESTs) were annotated by BLASTx and BLASTn in the Nr and Nt databases (unpublished). Two genes were annotated as ACO enzymes in safflower flower. Their missing 5′ fragment region and 3′ region were cloned through RACE, and the entire assembled sequences were labeled as CtACO1 and CtACO2, respectively. The cDNA of CtACO1 and CtACO2 both contained a 1098 bp open reading frame encoding a 365 amino acid with a predicted molecular weight of 40.8 kDa and a pI of 5.35. CtACO1 and CtACO2 were subjected to phylogenetic analysis with the ACOs of other plants (Figure S1). The sequence alignment showed that CtACO1 and CtACO2 were both characterized by the 2-oxoglutarate binding domain RxS and the ferrous iron binding site HxDxnH as in other ACOs, implying their physiologic function in the ethylene biosynthesis pathway, or at least as related to that of ACO (Figure 1).

### 2.2. Expression of CtACO1 and CtACO2 in Flowers at Different Flowering Times

**Figure 2:**
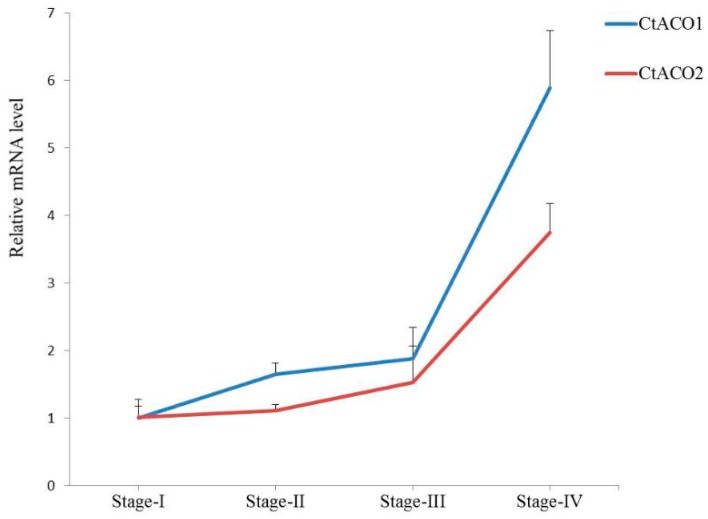
Expression patterns of CtACO1 and CtACO2 in flower stage I, stage II, stage III and stage IV.

To detect the expression patterns of CtACO1 and CtACO2 in flowers at different flowering times, flowers at stages I, II, III, and IV were collected [20]. As shown in Figure 2, the transcript levels of both CtACO1 and CtACO2 in flowers increased continuously with the elongation of the pollen tube. However, the transcript levels of CtACO1 in the four stages were all higher than those of CtACO2, especially in stage IV. Therefore, further investigations of CtACO1 were undertaken.

### 2.3. Subcellular Localization of CtACO1 Protein

**Figure 3:**
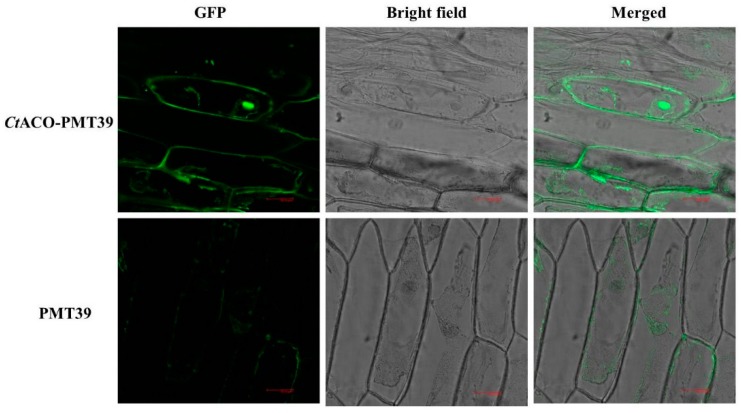
Subcellular localization of the CtACO1-PMT39 fusion protein in onion epidermal cells. The fluorescence emission signals were determined by a confocal laser scanning microscope. GFP fluorescence (GFP; green fluorescent protein), optical photomicrographs (bright field), and an overlay of bright and GFP fluorescence illumination (merged) are shown.

By using the WoLF PSORT prediction program for the subcellular localization of the CtACO1 protein, we predicted that the CtACO1 protein may be localized in the cytoplasm or nucleus. To examine this prediction, the recombinant of CtACO1 and pCAMBIA-1380-CaMV35S-MCS-EGFP-NOS (PMT-39) was introduced into onion epidermal cells through Agrobacterium strain LBA4404 mediation. The empty PMT-39 vector was also introduced into onion epidermal cells as a control. As shown in Figure 3, the CtACO1-PMT protein was preferentially localized in the cytoplasm and cytomembrane, whereas the PMT control emitted green fluorescent protein (GFP) signals in the cytoplasm. The combined results indicated that CtACO1 is a cytomembrane localized protein.

### 2.4. Transcript Expression Patterns of CtACO1 in Safflower

**Figure 4:**
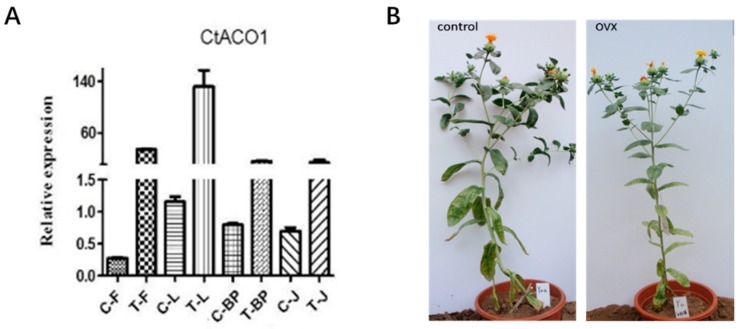
(**A**) Relative abundance of CtACO1 genes in flower (F), leaf (L), bracteole (BP) and stem (J) of control (C) and CtACO-overexpressing plants (T). (**B**) Phenotypic difference between WT (wild-type safflower line) and CtACO-overexpressing plants.

To explore the in vivo function of CtACO1, transgenic safflower that overexpressed CtACO1 under the CaMV 35S promoter was generated. Based on screening by genomic DNA PCR (Figure S2), 14 of 16 independent transgenic lines showed positive electrophoresis results. Therefore, six lines, namely numbers 5, 8, 9, 17, 18, and 19, were used for further analysis of the transcript levels of genes involved in the ethylene and flavonoid biosynthesis pathway. As shown in Figure 4A, compared with untreated safflower, the transgenic lines showed more than 20-fold enhanced CtACO1 transcript levels in leaf, bracteole, flower, and stem. Furthermore, there were almost no differences in plant morphology, growth and flower characteristics between the WT (wild-type safflower line) and the transgenic plants (Figure 4B).

### 2.5. Expression of Genes Involved in Ethylene and Flavonoid Biosynthesis Pathway

**Figure 5:**
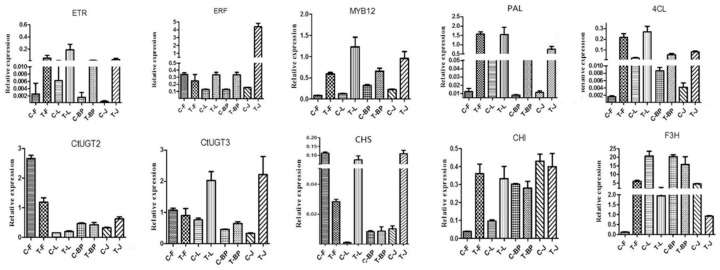
Relative abundance of genes involved in ethylene and flavonoid biosynthesis pathways in flower (F), leaf (L), bracteole (BP) and stem (J) of control (C) and CtACO-overexpressing plants (T).

As shown in Figure 5, ETR1 proteins in the ethylene signaling pathway could affect the flavonoid accumulation in plants, showing upregulation of transcript levels in overexpressed CtACO1 safflower in our study. The transcript levels of an immediate target for ETR, the ethylene-responsive gene (ERF), were significantly increased in the leaf, bracteole, and stem, but not in the flower. MYB12, which has been confirmed to control the flavonol biosynthetic genes in tissue- and cell-type-specific patterns, resulted in increased mRNA accumulation in the leaf, bracteole, flower, and stem in transgenic lines. To investigate the effects of CtACO1 overexpression on the flavonoid pathway, the transcript levels of genes encoding enzymes involved in flavonoid metabolism in the leaf, bracteole, flower, and stem were analyzed. The transcript abundance of 4CL, PAL, CHS, CHI, F3H, FNSI, F3’5’H, and glycosyltransferase (GT) in different organs showed different expression patterns in safflower lines with overexpressed CtACO1. As presented in Figure 5, the transcript levels of both 4CL and PAL in four organs of safflower lines with overexpressed CtACO1 showed more than 5-fold enhancement. However, the transcript levels of their downstream genes, including CHS, CHI, F3H, FNSI, F3’5’H, and UDP glycosyltransferase (UGT), differed among the organs. The transcript levels of 4CL, PAL, CHS, CHI, FNSI, F3’5’H, and UGT in the bracteole and stem of transgenic lines were all increased, whereas F3H showed decreased transcript levels compared with those in the bracteole and stem of controls. Except for FNSI and F3H, 4CL, PAL, CHS, F3′5′H, and two GTs in the leaf of transgenic lines showed enhanced mRNA accumulation. The flower is the medicament portion of safflower. The transcript levels of CHS, FNSI, and two UGTs in this organ were decreased due to the effect of CtACO1 overexpression. In contrast, the transcript levels of 4CL, PAL, CHI, F3H, and F3′5′H were positively regulated by the CtACO1 overexpression, increasing by more than 5-fold.

### 2.6. Metabolic Profile Analysis of Different Organs of Safflower

**Figure 6:**
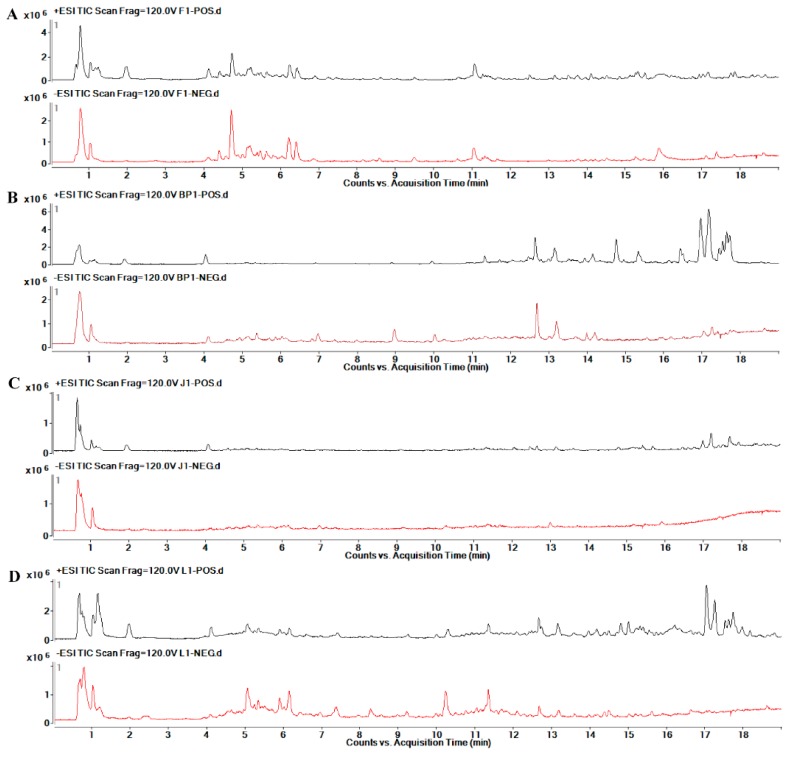
Representative total ion chromatograms (TICs) in electrospray ionization source (ESI) positive and negative ion mode from four different parts of safflower based on UHPLC-Q-TOFMS: (**A**) F, (**B**) BP, (**C**) J, (**D**) L.

**Figure 7:**
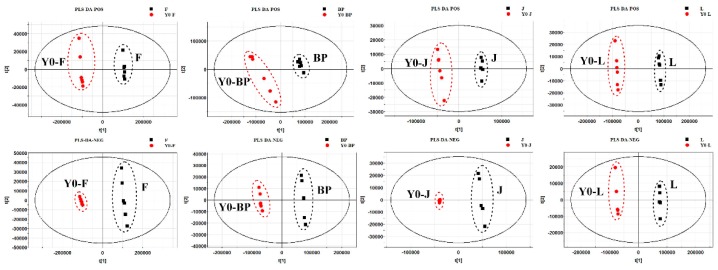
PLS-DA score plots of the transgenic group and control group in ESI positive and negative ion mode.

UHPLC-Q-TOF/MS was undertaken to analyze the four organs of safflower in eight groups; Figure 6 shows the representative total ion chromatograms (TICs) in the positive and negative modes. To characterize the metabolic differences of safflower between the transgenic and the control group, PCA, an unsupervised multivariate data analysis, was first done to visualize the trends and outliers in the data for the transgenic and control groups. The score plots showed that there were no outliers and that the transgenic group was clearly separated from the control group in both positive and negative modes, indicating that the metabolites from different safflower parts varied considerably between the groups. Then, supervised PLS-DA was applied to screen potential differential metabolites. As indicated by the PLS-DA score plots (Figure 7), the transgenic group was clearly separated from the control group. The cumulative R2X, R2Y, and Q2 were close to one, as shown in Table 1. The results of the permutation test showed no over-fitting in either the positive or the negative mode. PLS-DA scatter and variable importance plots can be used to identify characteristic metabolites to differentiate the transgenic and the control group. In this research, ions with VIPs greater than 1.5 and *p* < 0.05 were selected as important differential metabolites. Finally, 114 metabolites in the flower (F) part, 192 metabolites in the bracteole (BP) part, 245 metabolites in the leaf (L) part, and 113 metabolites in the stem (J) part, with significant differences between the transgenic and the control group, were identified.

### 2.7. Quantitative Detection of 12 Target Metabolites

**Figure 8:**
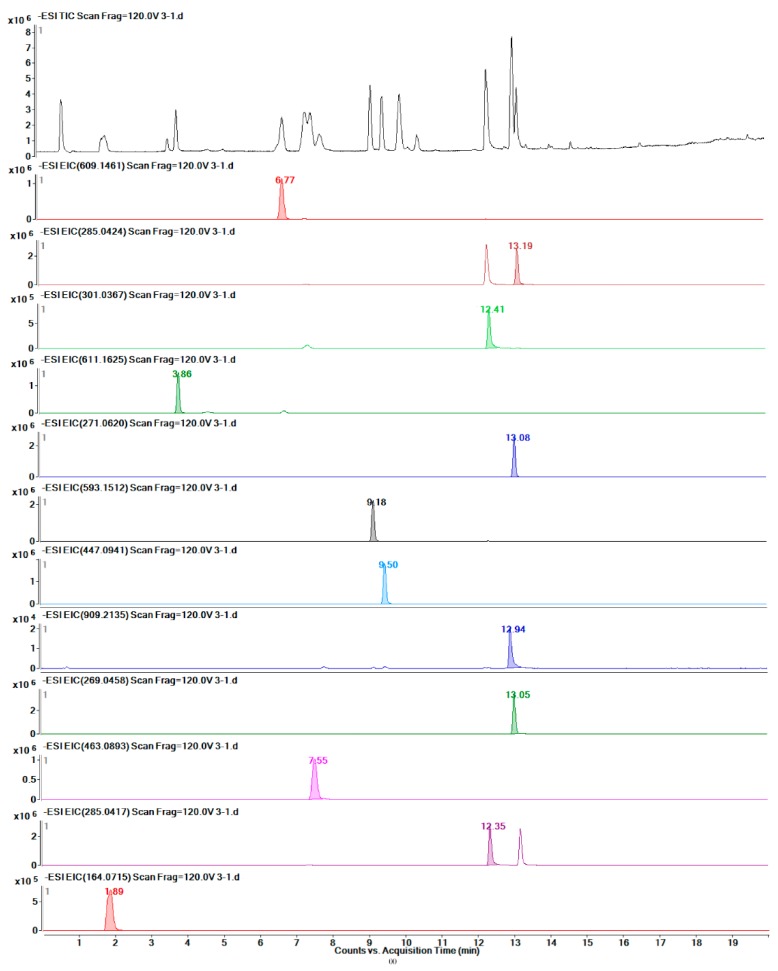
TIC of the mixed standards and EICs of each of the twelve metabolites.

Twelve target metabolites were identified by comparison with standards. The quantities of these metabolites were determined by applying LC-MS. Figure 8 shows the TICs of the total standards and of each metabolite.

**Figure 9:**
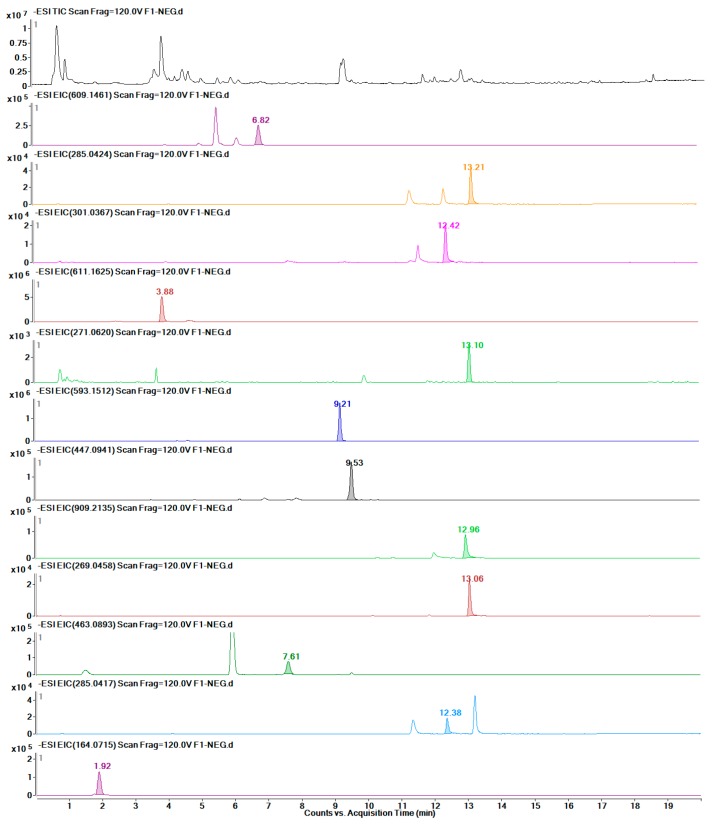
TIC of the F part and EICs of each of the twelve metabolites.

**Figure 10:**
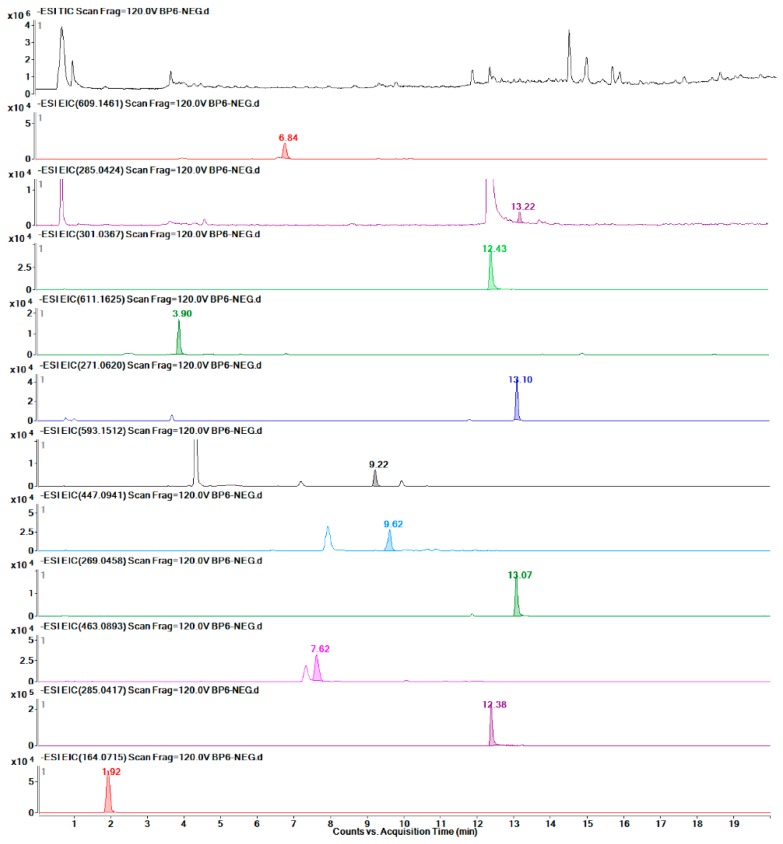
TIC of the BP part and EICs of each of the eleven metabolites.

**Figure 11:**
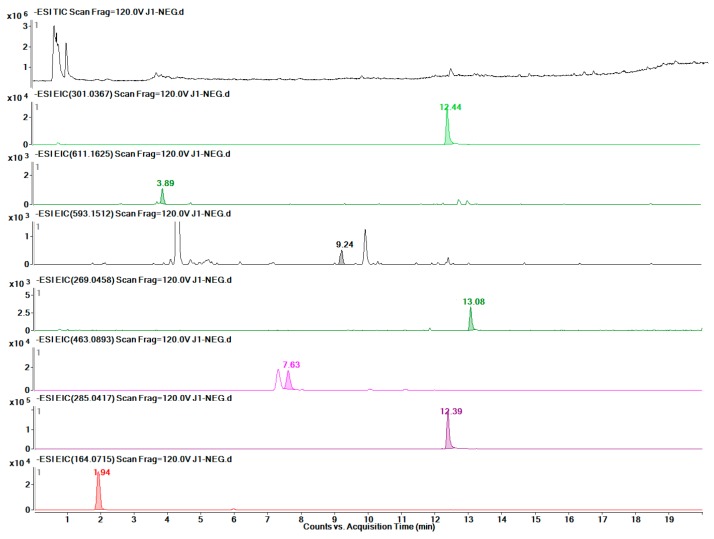
TIC of the J part and EICs of each of the seven metabolites.

**Figure 12:**
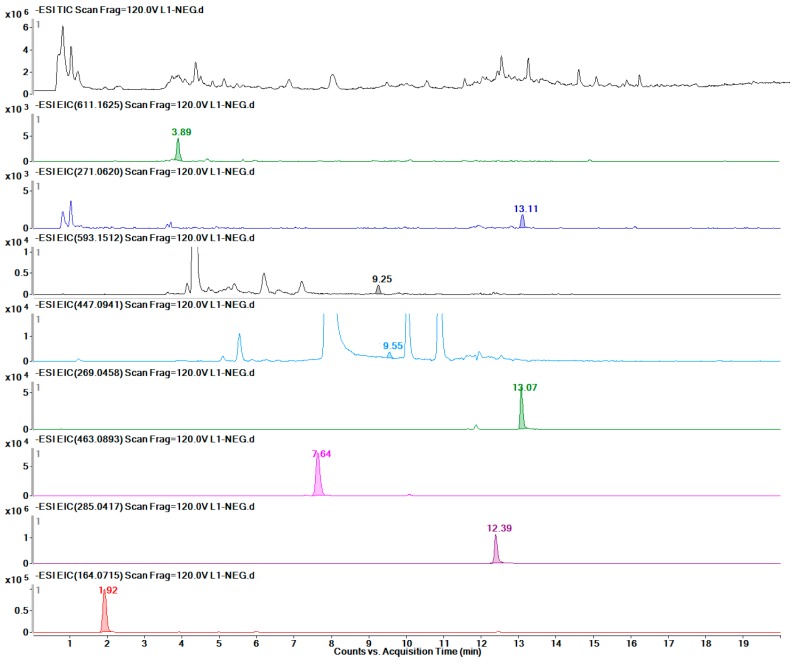
TIC of the L part and EICs of each of the eight metabolites.

As shown in Table 2, eight groups were detected by LC-MS, and the linear regression data of the investigated metabolites indicated that these quantitative detection methods had high stability. Finally, twelve metabolites were detected accurately in F (Figure 9), eleven in BP (Figure 10), seven in J (Figure 11), and eight in L (Figure 12). The data are presented in Table 3.

The changes of transcript abundance and compounds in the CtACO-overexpressing flower. The increased (with red up arrow) and decreased compounds (with black down arrow) are shown in the diagram. The upregulated genes are marked with a red up arrow and the downregulated genes are marked with a black down arrow.

## 3. Discussion

Flavonoids derived from phenylalanine and acetate metabolism are ubiquitously scattered across the plant kingdom and constitute various classes of metabolites. In the process of plant growth and development, flavonoids act as UV filters, signal molecules to the environment, visual and olfactory attractors to pollinators and herbivores, and protectors against light intensity, temperature, and biotic and abiotic stresses [21,22,23]. Therefore, the accumulation of flavonoids in plants is regulated by diverse developmental and environment cues. Hormonal regulation of the flavonoid biosynthetic pathway has been explored, with the results showing dissimilar functions in flavonoid accumulation in plants. Ethylene is a gaseous phytohormone that is implicated in countless and perplexing processes of plant growth and development. One of its effects on plant growth and development is the well-known “triple response” of etiolated dicotyledonous seedlings [24]. This response is characterized by depressant root elongation and exaggerated curvature of the apical hook. Enhanced ethylene levels have been reported to profoundly affect root gravitropism, which is believed to be mediated, at least partially, by flavonoid accumulation [25]. Meanwhile, ACC, the upstream precursor of ethylene, has been reported to be an inducer of flavonoid accumulation in the root tip. In the ethylene biosynthesis pathway, ACO acts as a pivotal regulatory enzyme that converts ACC to ethylene. Although ACO occurs solely in the ethylene biosynthesis pathway, it is widely constitutively present in most tissues [26].

In this study, two CtACOs from safflower were first cloned and identified. Similar to ACOs from other plants, CtACO1 and CtACO2 were characterized by the conserved 2-oxoglutarate and Fe (II)-dependent oxygenase superfamily domain. Bioinformation analysis suggested a physiologic function of CtACO1 and CtACO2 in the ethylene biosynthesis pathway. The transcript levels of CtACO1 in flower stages I, II, III, and IV were all higher than those of CtACO2. Regarding the amino acid level, CtACO1 was highly similar to CtACO2, with 99% identity. However, four amino acids were different; valine in position 21 of CtACO1 mutated into phenylalanine of CtACO2, serine in 208 into asparagine, arginine in 220 into cysteine, and asparagine in 362 into cysteine asparagine, which might lead to their dissimilar expression patterns at different flowering times.

At the cellular level, a previous study on tomato suspension cells showed that the location of ACO was a cytomembrane localized protein with cell fractionation technique [27]. However, Rombaldi et al. (1994), who used the immunocytolocalization method, showed that this enzyme was localized to the cell wall in the pericarp of ripening tomato and in climacteric apple [28]. Other later studies reported it as a cytosolic protein in the apple fruit pericarp tissue [29]. Here, by using electroporation transformation, CtACO1 from safflower was verified to be localized to the cytomembrane in onion epidermal cells, in accordance with a previous report [27]. These results suggest that ACO from different plants may present different subcellular localizations.

**Figure 13:**
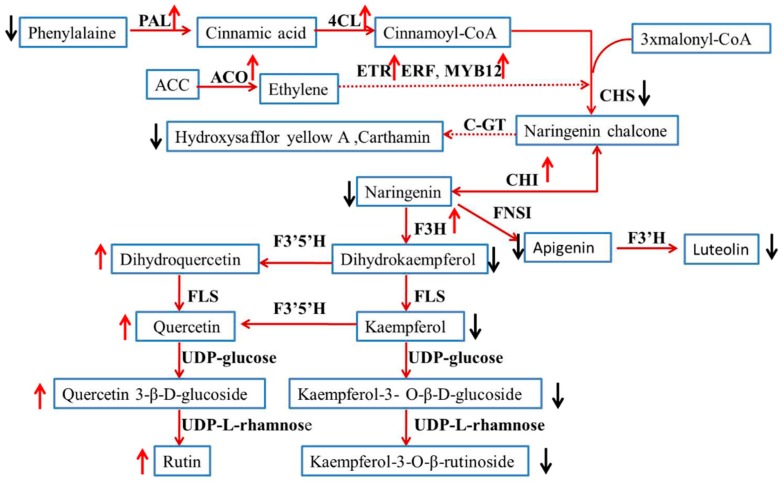
The presumed model dispalying the relationship between ethylene biosynthesis and flavonoid biosynthesis in safflower. Intermediate compounds are represented in the blocks. Enzymes involved in this study are shown in boldface.

The use of transgenic technology to alter plant characteristics has been shown to be a feasible way to quickly establish plants to improve the production of bioenergy crops [30]. Regarding the ACO gene function, after ACO transformation, most of the attention has been on its effect on the levels of climacteric ethylene and the rate of ripening, as studied in kiwifruit and papaya [31,32]. Genetic and molecular analyses of constitutive triple-response mutants, ethylene-insensitive mutants, and tissue-specific ethylene-insensitive mutants have shown that a largely linear ethylene response pathway is involved in transcriptional regulation in the nucleus [33]. As previously reported, the enzyme essential for flavonoid biosynthetic and pathway products accumulates in the cytoplasm and nucleus [34]. A later study pointed out that gene encoding enzymes of flavonoid metabolism may be the ultimate targets of ethylene transcriptional networks [35]. To test the hypothesis that CtACO1 could affect the flavonoid accumulation in safflower, transgenic safflower with overexpressed CtACO1 was generated. In this study, there was no typical phenotype change in the transgenic safflower with overexpressed CtACO1. However, the transcript abundance of genes encoding ethylene and flavonoid biosynthesis in these transgenic plants showed significant alteration. A model was constructed to synthesize the transcript abundance data, as shown in Figure 13. This model outlines the relationship between the ethylene biosynthesis and the flavonoid biosynthesis. Flowers are the main medicinal parts of safflower, which accumulates a striking variety of compounds. Figure 13 shows the changes in the detected genes and compounds. The transcript abundance of CtACO1 genes in overexpressed CtACO1 safflower was remarkably increased. Moreover, other genes related to the ethylene signaling pathway, including CtEin2 and CtETR1, showed upregulated transcript abundance. Regarding the effect of overexpressed CtACO1 on genes in flavonoid biosynthesis, varying effects between genes and metabolites were observed. Interestingly, although the transcript abundance of CtCHI was increased 9-fold in the overexpressed CtACO1 flower, the detected naringenin showed a 3-fold decrease. The explanation for this result may be that the metabolite biosynthesis is likely to lag behind the gene transcript expression events; thus, the enzyme synthesis, gene expression, and metabolite changes may be temporally dissimilar. Flavonols are among the types of flavone compounds in safflower. One surprising result from the transcript and metabolite analysis was that though the transcript abundance of CtF3H gene—a nuclear enzyme acting at the bifurcation of the flavonoid biosynthetic pathway—was enhanced by more than 40-fold in the flower after transgenic treatment, this coincided with kaempferol and its glycosylated derivatives (kaempferol-3-*O*-β-rutinoside and kaempferol-3-*O*-β-d-glucoside) displaying lower accumulation in the CtACO1-overexpressing flower. In contrast, the detected quercetin and its glycosylated derivatives (quercetin 3-β-d-glucoside and rutin) were increased in the flower. A previous report has shown that the different transcript expression ratios of the F3’H/CHS gene could led to disparate quercetin/kaempferol metabolite accumulation in tissue [36]. In our transcript analysis of safflower with overexpressed CtACO1, the fold increase of the F3’H gene affected by CtACO1 overexpression was considerably greater than that of CHS, which could have caused the increased accumulation of quercetin and its glycosylated derivatives (quercetin 3-β-d-glucoside and rutin). This report also showed that the ethylene-induced increases in flavonol accumulation, as measured by DPBA fluorescence, were most apparent in the elongation zone and thus well- positioned to contribute to root elongation and gravitropism. Additionally, the accumulation of apigenin and luteolin in different tissues affected by CtACO1 overexpression, especially their antipodal accumulation pattern in the leaf, was evidently dissimilar, which further enriched the potential effect of ethylene on apigenin derivatives.

Quinochalcones are considered to be the characteristic and major active constituents of safflower. Our previous study showed that the small heat shock protein from safflower might be involved in the formation of hydroxysafflor yellow A [37]. Thus far, the exact biosynthetic pathway of hydroxysafflor yellow A remains undetermined. Based on its chemical structure, we hypothesize that naringenin chalcone may be catalyzed by UDP-glycosyltransferases to generate hydroxysafflor yellow A. Similarly to the accumulation of kaempferol glycosylated derivatives, hydroxysafflor yellow A and carthamin in overexpressed CtACO1 safflower showed a markedly decreased accumulation compared to untreated safflower. This result suggested the negative regulation of overexpressed CtACO1 genes on the accumulation of quinochalcones from safflower, which lays the foundation for controlling the biosynthesis of hydroxysafflor yellow A. These results support the potential effect of ethylene on quinochalcones apart from derivatives of quercetin, kaempferol, and apigenin.

In sum, the floral dipping method was applied to generate transgenic safflower with overexpressed CtACO1. Meanwhile, the effect of CtACO1 overexpression on the transcript and metabolite levels in safflower was confirmed, which indicated varying effects between genes and metabolites. Taken together, the results of this study validated a novel regulatory effect of CtACO1 in the flavonoid biosynthetic pathway, thus providing hypothetical and practical groundwork for further work on regulating the overall metabolic flux of flavonoids in safflower, particularly hydroxysafflor yellow A and other quinochalcones, by applying appropriate genetic engineering strategies.

## 4. Materials and Methods

Discovery of CtACO from the transcription profiling database. To investigate the functional genes in safflower, flower transcriptome sequencing was done. The expressed sequence tags (ESTs) were annotated by BLASTx and BLASTn in the Nr and Nt databases (unpublished). Two genes were annotated as ACOs in safflower flower.

### 4.1. Plant Materials

A safflower line (No. 0025), identified as *Carthamus tinctorius* L. by Professor Meili Guo, was planted in the greenhouse at the School of Pharmacy in the Second Military Medical University (Shanghai, China). The voucher specimen was SMMU141226 and was deposited at the Medicinal Plant Herbarium of the Department of Pharmacognosy, School of Pharmacy, Second Military Medical University.

The safflower line No. 0025 was grown in the greenhouse of the Second Military Medical University (Shanghai, China). The plants were maintained at a mean temperature of 25 °C over a photoperiod of 16 h.

### 4.2. Analysis of the Transcription Profiling Database of Safflower

To decrease the gene copy variation at the transcription level, normalized cDNA libraries of safflower flowers at different developmental stages were constructed. Then, safflower transcriptome sequencing was undertaken to detect more genes related to the biosynthetic pathway of the active components. The genes were annotated by BLASTx and BLASTn search homologs in the Nr and Nt databases. Two ACOs were selected from the annotated genes.

Isolation and cloning of CtACOs. To obtain the full-length cDNA sequence of CtACOs in *Carthamus tinctorius* L., 5′- and 3′-RACE experiments were carried out by using the SMART-RACE cDNA amplification kit (Clontech, Mountain View, CA, USA). Gene-specific primers denoted as GSP-1/-2 (Table S1) were designed. Amplified fragments of 5’- and 3’-RACE were cloned in the pMD-19 vector (Takara, Dalian, China) for sequencing. The primer pairs ACO1-F/-R and ACO2-F/-R were subsequently designed, based on the result of the sequence assembly (Table S1), to amplify the full-length cDNA of CtACO1 and CtACO2. The amplified fragment was cloned into the pMD-19 vector and then sequenced.

### 4.3. Sequence Alignment and Phylogeny Analysis

The respective sequences of the CtACO1 and CtACO2 mRNA have been submitted to GenBank under accession numbers KU291182 and KU291190. The theoretical isoelectric points and mass values for the proteins were predicted using the ExPASyProtParam tool (http://web.expasy.org/protparam/). The CtACO1 and CtACO2 protein sequences were aligned with the protein sequences of ACO genes from other plants through the National Center for Biotechnology Information (NCBI: http://www.ncbi.nlm.nih.gov/). To identify the conserved motifs of the CtACO1 and CtACO2 proteins, their deduced amino acid sequences were aligned with ACOs from other plants using DNAMAN 8.0 software (Lynnon Biosoft, San Ramon, CA, USA). Phylogenetic trees were constructed with the MEGA 5.10 program using the neighbor-joining (NJ) method and 1000 bootstrap replicates [38].

### 4.4. Subcellular Localization of the CtACO1 Protein

Subcellular localization of the CtACO1 protein was first done using the online prediction program WoLF PSORT (http://www.genscript.com/psort/wolf_psort.html). To further identify the outcomes of the prediction, the recombinant of CtACO1 and pCAMBIA-1380-CaMV35S-MCS-EGFP-NOS (PMT-39, modified by our laboratory) vector was constructed with specific primers (Table S1). Then, the amplified fragment was infused with the linearized PMT-39 vector digested by Hind III and BamH I with the use of the SunBio TM cloning kit (SunBio, Shanghai, China). After verification through sequencing, the overexpressing recombinant plasmid and PMT-39 were transformed into A. tumefaciens strain LBA4404 electrocompetent cells (Takara Bio, Shiga, Japan) by using an electroporation apparatus. Positive monoclonal Agrobacterium was chosen and cultured in Luria-Bertani (LB) medium supplemented with 50 mg/L kanamycin and 100 mg/L streptomycin. When the value of OD600 was about 1.0, Agrobacterium was centrifuged at 5500 rpm for 10 min and resuspended with the same volume of Murashige and Skoog (MS) liquid medium. The prepared onion epidermal layers were placed in the MS liquid medium for 20 min, with gentle shaking every 5 min, and then incubated on MS solid agar with 0.4 mol/L mannitol at 25 °C in the dark for 24 h. The GFP fluorescence of the CtACO protein and PMT-39 were observed under a confocal microscope (Leica TCS SP5).

### 4.5. Transformation of Safflower and Screening

Agrobacterium tumefaciens strain LBA4404 harboring the above overexpressing recombinant of CtACO and PMT-39 vector was introduced into safflower plants using the floral dip method to generate safflower plants with overexpressing CtACO. For the construction of the CtACO1 overexpression vector, the CDS of CtACO1 was fused to a GFP tag and cloned into PMT-39 vector downstream of 35S promoter [20]. Then, the young euphylla tissues of T1 transformants were taken for initial screening analyses. The total genomic DNA of young euphylla tissues was extracted with Column Plant DNAout kits (QIAGEN, Hilden, Germany) and then analyzed by PCR with the use of specific primers labeled PMT-39F and CtACO-R (Table S1). The PCR system used 150 ng of total genomic DNA, 1 × PCR buffer for KOD FX, 0.2 mM dNTP, 0.5 μM primers, and 1 U of KOD FX (TOYOBO, Shanghai, China). The thermal profile of the PCR was as follows: initial denaturation at 94 °C for 2 min and then 40 cycles at 98 °C for 10 s, at 56 °C for 30 s, at 68 °C for 45 s, and, finally, at 68 °C for 2 min. After amplification, the PCR product was detected by electrophoresis on 1.0% *w*/*v* agarose gel in TAE buffer. Positive transgenic plants were grown to flower stage and used for further analysis.

### 4.6. RNA Isolation and qPCR

Regarding the analysis of CtACO1 and CtACO2 expression in the flower at different flowering times, flowers at stage I (the first day when the corolla protruded from the sepals), stage II (the second day when the corolla protruded from the sepals), stage III (the third day when the corolla protruded from the sepals), and stage IV (the fourth day when the corolla protruded from the sepals) were collected. For the qPCR analysis of transgenic safflower that overexpressed CtACO1, 3-day-old flowers of transgenic and control plants, as well as tissues of the leaf, stem, and bracteal leaf, were harvested in the same period. Total RNA was extracted from plants using TransZol reagents (TransGen Biotech, Shanghai, China) according to the manufacturer’s instructions. The RNA integrity was examined by running on 1.0% (*w*/*v*) agarose gel and by detecting the A260/280 ratio. RNA quantification was done on a Biomate 3S spectrophotometer (Thermo Scientific, Rockford, IL, USA). Subsequently, first-strand cDNA was synthesized using the TransScript One-Step gDNA Removal and cDNA Synthesis SuperMix kit (TransGen Biotech, Shanghai, China). cDNAs were diluted five times and then mixed with TransStart Green qPCR SuperMix (TransGen Biotech, Shanghai, China) and 0.4 uM of each primer for amplification. Table S1 shows the primers used. qRT-PCR was done with the ABI 7500 Real-Time PCR detection system (Applied Biosystems, Foster City, CA, USA). The PCR procedure was as follows: holding for 3 min at 95 °C, followed by 40 cycles of denaturation for 10 s at 95 °C, annealing for 20 s at 58 °C, and elongation for 30 s at 72 °C. The specificity of the amplification was checked by analyzing the melting curves and its procedure was 60 °C for 1 min and 95 °C for 30 s. The relative expression levels of genes in at least two replicates of safflower samples were all normalized to Ct60s (KJ634810) as the reference gene and then compared with the expression levels of the control by using the 2^−∆∆Ct^ method [39].

### 4.7. Metabolite Extraction and UHPLC-MS/TOF Analysis

Chemicals and reagents: Chromatography grade methanol and acetonitrile were purchased from Merck (Darmstadt, Germany). High liquid chromatography grade formic acid was purchased from Fluka (Buchs, Switzerland). Ultrapure water was prepared using a Milli-Q water purification system (Millipore Corp., Billerica, MA, USA). The following standard compounds were obtained from Aladdin: rutin, kaempferol, quercetin, apigenin, quercetin 3-β-d-glucoside, luteolin, d-phenylalanine, and hydroxysafflor yellow A. Narigenin was obtained from Sigma-Aldrich (St. Louis, MO, USA). Kaempferol-3-*O*-β-rutinoside, kaempferol-3-*O*-β-d-glucoside, and carthamin were synthesized in our laboratory. All other chemicals were of analytical grade, with purities higher than 98%.

### 4.8. Preparation of Sample

Before the analysis, the whole dried sample was ground to powder by using a disintegrator. Next, 1000 mL of 60% methanol was added to the powder (5 mg), which was then soaked overnight. After treatment by ultrasound for 40 min and passing through micropore filters, the clear supernatant (150 μL) was transferred into a sampling vial for UHPLC–MS analysis.

### 4.9. UHPLC-Q-TOF/MS Profiling Analysis of Different Safflower Parts between the Transgenic and Control Groups

A metabolomics approach that uses ultra-performance liquid chromatography coupled with quadrupole time-of-flight mass spectrometry (UHPLC-Q-TOF/MS) was developed to perform four different parts of safflower—F, BP, J, and L—metabolic profiling analysis and search for differential metabolites in the transgenic group and control group. On this basis, the present research explored the metabolic effect and regulation of ACO overexpression on the flavonoid metabolism of safflower.

UHPLC-Q-TOF/MS analysis was carried out on an Agilent 6538 Accurate Mass Quadrupole Time-of-Flight mass spectrometer (Agilent, Santa Clara, CA, USA). Chromatographic separations were done at 25 °C on an XBridge TM BEH C18 column (2.1 mm × 100 mm, 2.5 μm; Waters, Milford, MA, USA). The mobile phase consisted of 0.1% formic acid (A) and ACN modified with 0.1% formic acid (B); the post time for equilibrating the system was 6 min, and the flow rate was set to 0.4 mL/min. The optimized UPLC elution conditions were: 0–2 min, 5% B; 2–4 min, 5–20% B; 4–6 min, 20–21% B; 6–9 min, 21–26% B; 9–10 min, 26–40% B; 10–15 min, 40–80% B; 15–17 min, 80–95%; and 17–19 min, 95% B. An electrospray ionization source (ESI) was operated in both positive and negative modes. The optimized conditions were as follows: capillary voltage, 4 kV in positive mode and 3.5 kV in negative mode; drying gas flow, 11 L/min; gas temperature, 350 °C; nebulizer pressure, 45 psig; fragmentor voltage, 120 V; and skimmer voltage, 60 V. The mass spectrum was collected in centroid mode, ranging from 50 to 1100 *m*/*z*. The reference ions were 121.0509 and 922.0098 in positive mode, and 119.03632 and 966.000725 in negative mode.

All data were acquired using the Agilent MassHunter Workstation B.04.00 analysis software. The MS data were determined, extracted, and integrated with the Agilent Profinder B.06.00 software. All data were weight-normalized using Excel. The three-dimensional data matrix, including the retention times, *m*/*z* data, and normalized ion intensities, was imported into the SIMCA-P program (Umetrics, Umea, Sweden) for multivariate statistical analysis to screen the differential metabolites.

### 4.10. Quantitative Determination of Target Differential Metabolites

After analyzing and fitting the samples from four different parts of the transgenic and control groups using metabolomics, liquid chromatography tandem mass spectrometry was used to detect the accurate concentrations of target metabolites in the four parts of these groups.

### 4.11. Chromatography and Mass Spectrometry

The column was kept at a temperature of 25 °C, and the flow rate was 0.4 mL/min, with an injection volume of 4 μL. The UHPLC binary solvent system consisted of mobile phases A (0.1% formic acid) and B (ACN modified with 0.1% formic acid). The gradient elution UHPLC conditions were: 0–2 min, 5% B; 2–2.5 min, 5–15% B; 2.5–7.5 min, 15% B; 7.5–8 min, 15–20% B; 8–10 min, 20–21% B; 10–18 min, 21–95% B; and 18–20 min, 95% B; the system was equilibrated with a post time of 5 min. The 12 components were measured well in the negative mode. Thus, the negative mode was used to analyze the differences. The mass spectrometry method was the same as previously described.

### 4.12. Preparation of Standards

The stock solutions of the standards were resolved with 60% methanol. The concentrations of the standards were as follows: naringenin, 1.36 mg/mL; rutin, 1.32 mg/mL; quercetin, 1.04 mg/mL; kaempferol, 1.11 mg/mL; kaempferol-3-*O*-β-rutinoside, 1.67 mg/mL; kaempferol-3-*O*-β-glucoside, 1.04 mg/mL; apigenin, 1.06 mg/mL; HSYA, 1.3 mg/mL; isoquercitrin, 1.23 mg/mL; luteolin, 1.05 mg/mL; d-phenylalanine, 3.72 mg/mL; and carthamin, 0.266mg/mL. The same volume of standard solutions was mixed and titrates ratio diluted. l-para-chlorophenylalanine was added as an internal standard, with a final concentration of 10 μg/mL.

### 4.13. Validation of the Quantitative Measurement Method

The standard curve of 12 objective compounds was drawn. The accuracy and limit of quantification were evaluated.

### 4.14. Quantitative Measurement of Objective Metabolites

The internal standard was added to the samples at the same concentration. The above-mentioned chromatography and mass spectrometry methods were used to detect metabolites in the samples from four parts of the transgenic and control groups. Agilent Profinder B.06.00 software (Agilent Technologies, Santa Clara, CA, USA) was applied to complete the peak extraction and integration. Students *t* test was used to determine the statistical significance.

## Figures and Tables

**Table 1:** R^2^X, R^2^Y and Q^2^ values of PLS-DA in both positive and negative modes.

Parameter	F		BP		J		L	
	POS	NEG	POS	NEG	POS	NEG	POS	NEG
R^2^X	0.97	0.979	0.86	0.899	0.857	0.979	0.891	0.927
R^2^Y	1	0.999	0.998	1	0.998	0.999	1	1
Q^2^	1	0.999	0.995	0.999	0.993	0.999	0.998	0.999

**Table 2:** Linear regression data of the investigated metabolites.

No.	Analytes	Linear Regression			LOQ (ng/mL)	Precision (RSD%, n = 6)
		Regression Equations	Linear Ranges (ng/mL)	R^2^		
1	Rutin	y = 0.3535x − 0.0059	20.3–5202.7	0.9997	20.3	1.56
2	Kaempferol	y = 0.9063x + 0.0043	4.3–273.4	0.9985	4.3	1.42
3	Quercetin	y = 0.3528x + 0.0142	16–1024.8	0.9951	16	1.86
4	Hydroxysafflor yellow A	y = 0.2511x − 0.0167	20–20495.5	0.9999	20	2.49
5	Naringenin	y = 0.9582x + 0.0057	2.6–335	0.9967	2.6	0.66
6	Kaempferol-3-*O*-β-rutinoside	y = 0.4554x + 0.001	6.4–6582.2	1	6.4	1.34
7	Kaempferol-3-*O*-β-d-glucoside	y = 0.5133x − 0.0006	4–4099.1	0.9999	4	1.26
8	Carthamin	y = 0.0245x + 0.0013	262.1–16774.8	0.9999	262.1	3.37
9	Apigenin	y = 1.45x + 0.0065	2–261.1	0.9971	2	1.43
10	Quercetin 3-β-d-glucoside	y = 0.4194x + 0.0055	18.9–1212	0.9994	18.9	1.43
11	Luteolin	y = 1.2568x + 0.0248	2–1034.6	0.9958	2	0.92
12	Phenylalanine	y = 0.0925x + 0.0176	57.3–14662.2	0.9984	57.3	0.83

**Table 3:** The quantitative determination results of 12 metabolites from 4 different parts of safflower.

Part	The Contents of Flavone in Different Organs (%)											
	Rutin	Kaempferol	Quercetin	Hydroxysafflor Yellow A	Naringenin	Kaempferol-3-*O*-β-rutinoside	Kaempferol-3-*O*-β-d-glucoside	Carthamin	Apigenin	Quercetin 3-β-d-glucoside	Luteolin	Phenylalanine
F	0.0707	0.0032	0.0047	0.8289	0.0002	0.3898	0.0217	0.4125	0.0012	0.0151	0.0011	0.1654
Y0-F	0.0401	0.0154	0.0024	1.7159	0.0005	0.6276	0.0598	1.301	0.0036	0.0087	0.0013	0.2593
FC	1.76	0.21	1.93	0.48	0.30	0.62	0.36	0.32	0.34	1.72	0.84	0.64
P	9.30 × 10^−5^	2.38 × 10^−8^	3.73 × 10^−3^	5.39 × 10^−7^	7.68 × 10^−5^	5.50 × 10^−6^	3.85 × 10^−9^	1.29 × 10^−4^	3.69 × 10^−6^	8.19 × 10^−4^	5.02 × 10^−1^	2.08 × 10^−3^
BP	0.0083	0.0001	0.0094	0.0084	0.003	0.0015	0.0057	——	0.0009	0.0098	0.0137	0.0796
Y0-BP	0.0019	0.0007	0.0001	0.0216	0.0032	0.0039	0.01		0.0002	0.0003	0.0033	0.1929
FC	4.44	0.18	68.3	0.39	0.93	0.39	0.57		4.74	29.38	4.13	0.41
P	5.64 × 10^−5^	3.36 × 10^−3^	1.27 × 10^−3^	9.23 × 10^−5^	5.76 × 10^−1^	2.56 × 10^−5^	4.78 × 10^−4^		1.66 × 10^−3^	4.03 × 10^−7^	1.26 × 10^−4^	2.26 × 10^−6^
J	——	——	0.0038	0.0019	——	0.0001	——	——	0.0002	0.0064	0.0149	0.0435
Y0-J			0.0083	0.0063		0.0007			0.0001	0.0049	0.0142	0.0565
FC			0.46	0.31		0.1			2.26	1.31	1.05	0.77
P			4.29 × 10^−3^	1.82 × 10^−6^		1.57 × 10^−5^			1.21 × 10^−1^	3.40 × 10^−2^	7.44 × 10^−1^	8.47 × 10^−2^
L	——	——	——	0.0019	0.0001	0.0003	0.0002	——	0.002	0.0129	0.0711	0.066
Y0-L				0.006	0.0001	0.0007	0.0002		0.0033	0.0011	0.0051	0.0717
FC				0.31	0.46	0.42	0.9		0.59	12.06	13.95	0.92
P				3.52 × 10^−9^	1.66 × 10^−2^	3.69 × 10^−5^	4.60 × 10^−1^		1.92 × 10^−2^	7.77 × 10^−4^	2.39 × 10^−5^	2.76 × 10^−1^

## Supplementary Materials

The following are available online. Figure S1. Phylogenetic tree of amino acid sequences of ACOs from different plant species. The tree was constructed using MEGA 5.0 with 1000 bootstrap replicates. The length of the lines represents the relative distance between nodes. Genbank accession numbers amino acid sequences of ACOs used for construction of the tree were as follows: SlACO1(XP_004236932), StACO11 (XP_006355064), SpACO 4(XP_015074312), CaACO4(XP_016572720), TcACO (XP_007045252), GaACO(XP_017612243), GhACO(XP_016665668), RcACO(XP_002525989), CtACO1(KU291182), CtACO2(KU291190). PmACO(XP_008221470) and NtACO11(XP_016495862). Figure S2. Agarose Gel Electrophoresis analysis of PCR product amplified with genomic DNA of young euphylla tissues from T1 safflower plants. M: DNA marker, 1–3: PCR product amplified with genomic DNA of safflower harbored PMT39 plasmid, PMT39 plasmid and CtACO-PMT39 plasmid. 4–19: PCR product amplified with genomic DNA of young euphylla tissues from T1 safflower plants. Table S1. Primer information for the present investigation.

## Abbreviations

ACO	1-aminocyclopropane carboxylic acid oxidase
GFP	green fluorescent protein
PMT-39	pCAMBIA-1380-CaMV35S-MCS-EGFP-NOS
LB	Luria-Bertani
MS	Murashige and Skoog
UHPLC-Q-TOF/MS	Ultra performance liquid chromatography-quadrupole-time-of-flight massspectrometry
F	flower
L	leaf
BP	bracteole
J	stem
C	control
