# Supplementary figures and images for: CtACO1 Overexpression Resulted in the Alteration of the Flavonoids Profile of Safflower
Source: Molecules. 2019 Mar 21;24(6):1128. doi: 10.3390/molecules24061128 (PMC6471848; doi:10.3390/molecules24061128)

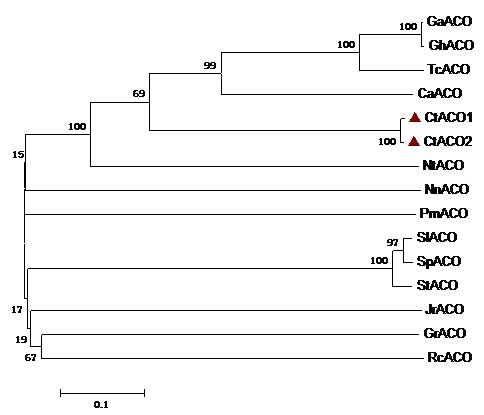

Supplement: Supplementary file 1 [file molecules-24-01128-s001.zip › Additional file/Additional file 1.jpg]

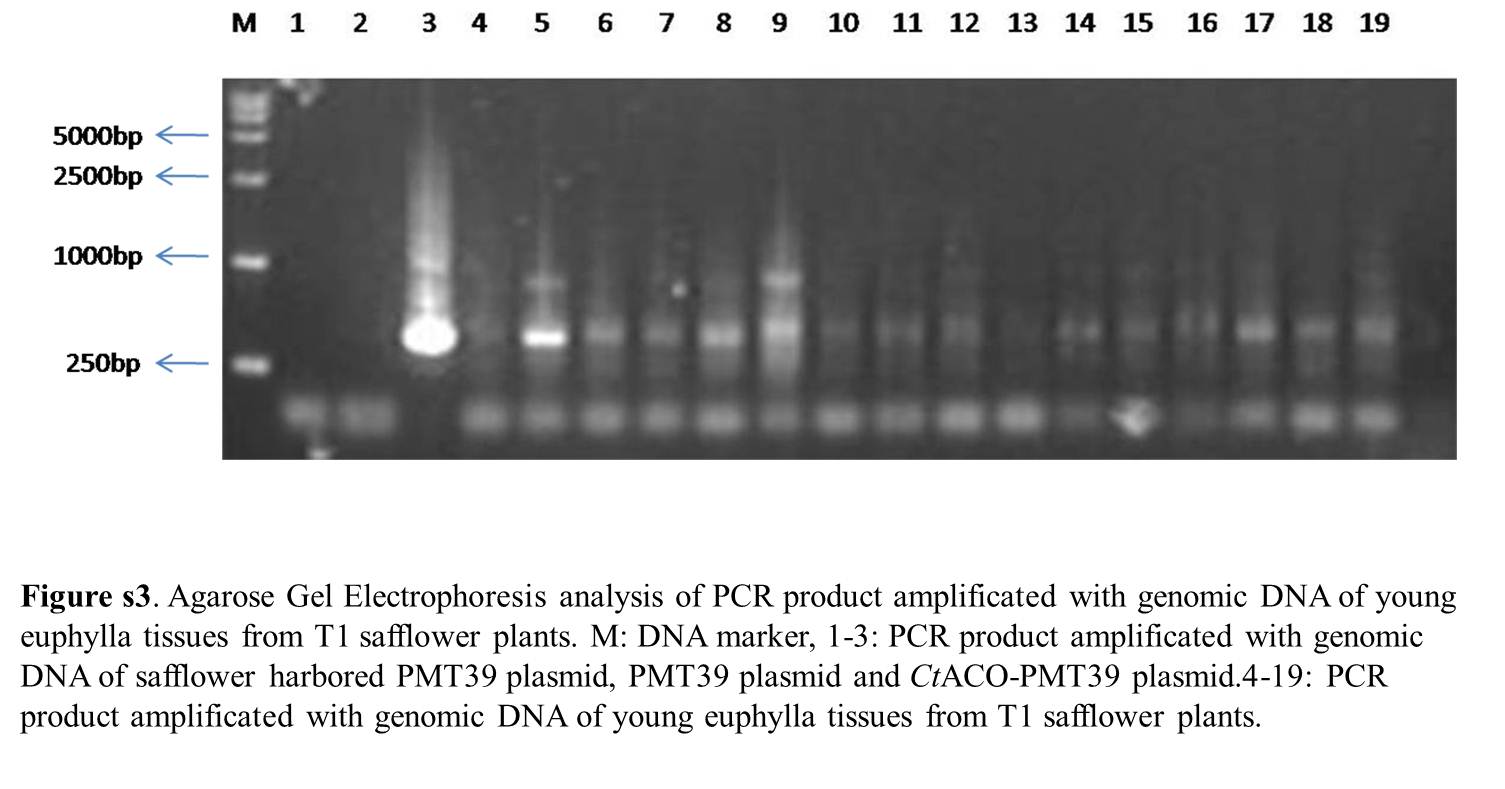

Supplement: Supplementary file 1 [file molecules-24-01128-s001.zip › Additional file/Additional file 2.jpg]
