# Supplementary material for: CtACO1 Overexpression Resulted in the Alteration of the Flavonoids Profile of Safflower
Source: Molecules. 2019 Mar 21;24(6):1128. doi: 10.3390/molecules24061128 (PMC6471848; doi:10.3390/molecules24061128)
Supplement: Supplementary file 1 [file molecules-24-01128-s001.zip › Additional file/Table S1.doc]

**Table S1.** Primer information for the present investigation.

| **Gene** | **Forward and reverse primers** | **Purpose** |
| --- | --- | --- |
| CtACO1 | GSP1:ATGGTGGTTAGGCCGGAATCTAGCAGTC  GSP2:GACTGCTAGATTCCGGCCTAACCACCAT | 5’RACE and 3’RACE |
| CtACO2 | GSP1: TGACCTGCAACAACCCGCCGACCTC  GSP2: TCCCATTACTATCCCCAGTGCCCACAGCC | 5’RACE and 3’RACE |
| CtACO1 | F:CGAATCTAGACCGTCAGCCATG  R:GCATCCATGGTCGACTCAACGC | full-length cDNA sequence PCR |
| CtACO2 | F: CGAATCTAGACCGTCAGCCATG  R: GCATAGATGGTCGACTCAACGC | full-length cDNA sequence PCR |
| CtACO1 | F: ATGATGTCTAACGACGAA  R: CGGTCCATATAGGTTTTC | qRT-PCR |
| CtACO2 | F: CATAGAGTGCTGGCTAACCC  R: TTCTCGGATGCTTGGATTGA | qRT-PCR |
| CtACO1 | F:GAGCTTTCGCGGATCCGCCACCATGGCTTCAGCCACTAACGAAC  R1:CACGTCACCGCATGTTAGAAGACTTCCTCTGCCCTCAGCATTCGTGTCATCGATTCTG  R2:CATGGTGGCAAGCTTAGGGCCGGGATTCTCCTCCACGTCACCGCATGTTAGAAG | Overexpression vector construction |
| CtACO1 | F1:CGAACCACACAGCAGCTACGATC  R1:CATGGTGGCAAGCTTAGGGC  F2:ATCTCTCTCGAGCTTTCGCGG  R2:CGTCGCCGTCCAGCTCGACCAG | Recombinant genomic DNA PCR |
| CtACS | F:ATAAAGGCGTTTGTGCTGAA  R:TCCGAGACGATAACTTGAGAT | qRT-PCR |
| AtEIN2 | F:CTTCTGGCAATACTGATACT  R:GACATAGACAATCCTGAGATAT | qRT-PCR |
| At ETR1 | F:ATACACTTCGTCATCAACATC  R:TAGCCACAGGAGAATTAGGA | qRT-PCR |
| ERF6 | F:CAGTTCCGCCATTGAAGTTG  R:TTTCCTCTACCGCCTCTTTG | qRT-PCR |
| MYB 12 | F:CTGATTCGGTTGCTGCTGTG  R:TGCTGTAGAGATGGTTGAAGAAGA | qRT-PCR |
| GmPAL | F:CAACCAGGATGTCAACTCTT  R:GCAATCAAGAATGTGGAAGAC | qRT-PCR |
| Gh4CL | F:AGCAAGTGGATGGAGAGAAC  R:CTGTTGAGTGAGTAAATGTGGAAT | qRT-PCR |
| CtCHS | F: CACCTTCTGAAAGATGTT  R: GTTCCAATCCGATACC | qRT-PCR |
| CtCHI | F: ACTGGTGTTATGGTAGGA  R: GGATACTTCAAGGTAAGGATA | qRT-PCR |
| CtF3H | F:ACACGAACCGACTATCCATA  R:GACCTATCTCCTTCATTCACTT | qRT-PCR |
| CaF6H | F:TGAAGGATAACGGATGGATA  R:TGTATGCTTGATGAGACTTG | qRT-PCR |
| VvF3’H | F: CTCACGAAGAAATGGATCAA  R: ATATGGCTTGTAGGTATGGA | qRT-PCR |
| BmFNSI | F:GATTACGGAACCTTCACGCT  R: CCACTTCTCGCTACCATTCA | qRT-PCR |
| CtUGT1 | F: GGAAGAGGAGTATGATGA  R: AATGGCAGTTGAGATTAC | qRT-PCR |
| CtUGT2 | F: CTACACCAACCTCAATCG  R: AGTCACATCCACATTCAAG | qRT-PCR |
